# Gastric Outlet Obstruction Caused by a Duodenal Clot

**DOI:** 10.7759/cureus.22814

**Published:** 2022-03-03

**Authors:** Tavia Buysse, Vikram Kotwal

**Affiliations:** 1 Internal Medicine, Rush University Medical Center, Chicago, USA; 2 Gastroenterology, John H. Stroger, Jr. Hospital of Cook County, Chicago, USA

**Keywords:** pyloric ulcer, gastric distention, nsaid induced gastritis, complicated peptic ulcer disease, esophago-gastro-duodenoscopy, upper gastro-intestinal bleed

## Abstract

Gastric outlet obstruction (GOO) is a mechanical obstruction usually located in the gastric pylorus or duodenum. After the introduction of proton pump inhibitors (PPIs) in the late 1980s, most cases of gastric outlet obstruction are now caused by malignancy and peptic ulcer disease rarely leads to obstruction. We present a case of GOO caused by a large clot in the pylorus, preventing visualization of the source of bleeding. As the removal of the obstructing clot was deemed too high risk, the patient was treated with promotility agents that relieved the obstruction and allowed for the identification of the etiology of his upper gastrointestinal bleeding. Bleeding was definitively managed with embolization of the gastroduodenal artery.

## Introduction

Gastric outlet obstruction (GOO) is a mechanical obstruction usually located in the gastric pylorus or duodenum that typically presents with abdominal pain and postprandial vomiting [1]. Before the advent of proton pump inhibitors, 90% of all cases of GOO were caused by peptic ulcer disease (PUD). Currently, 50-80% of GOO cases are due to malignancy and obstruction is considered a rare complication of PUD [2-6].

## Case presentation

**Figure 1:**
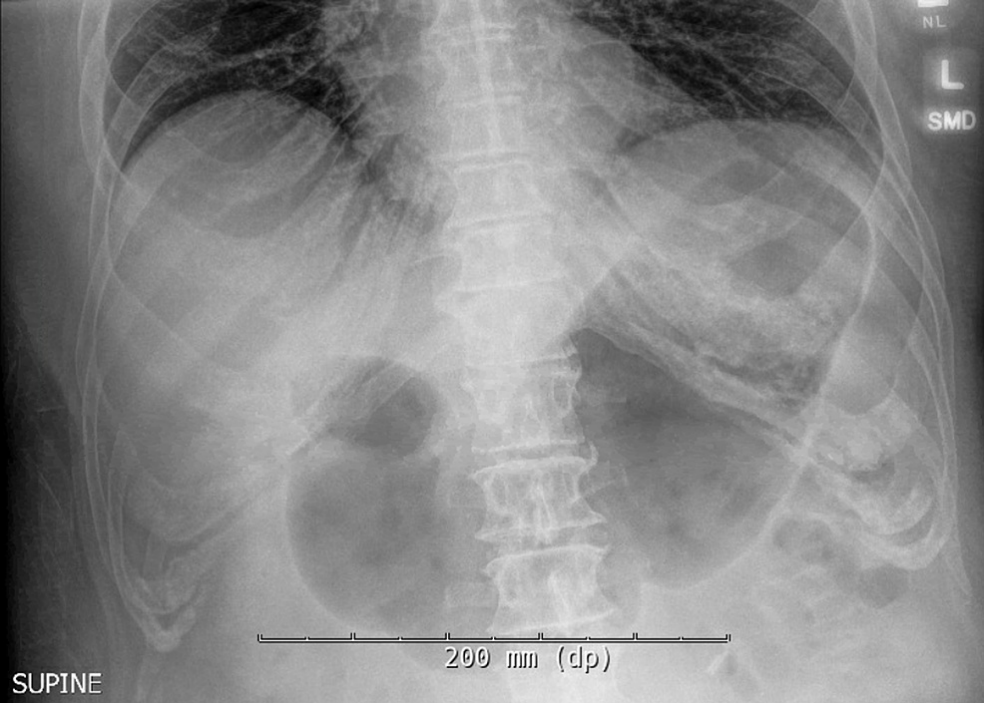
Abdominal x-ray demonstrating gastric distension.

**Figure 2:**
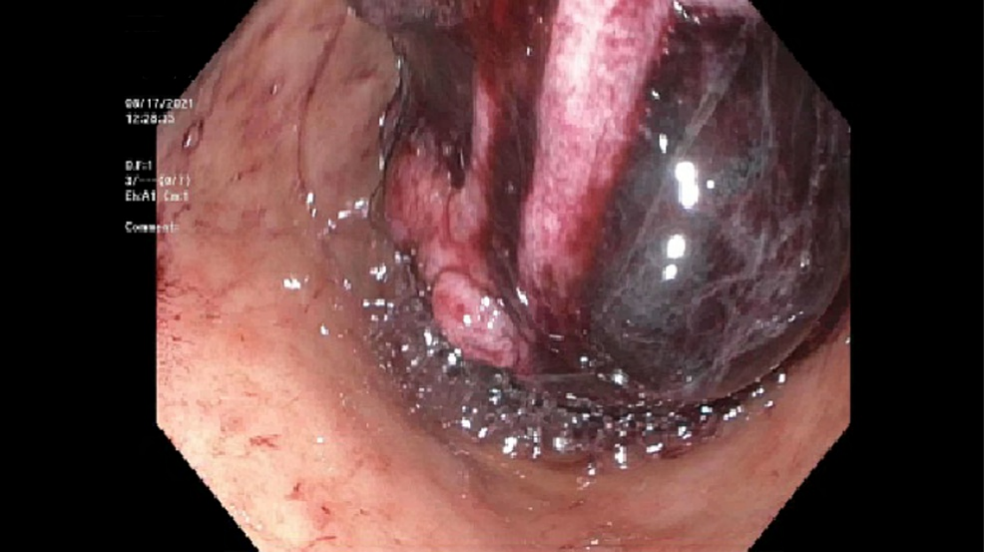
Blood clot obstructing the pylorus during initial Esophagogastroduodenoscopy (EGD).

**Figure 3:**
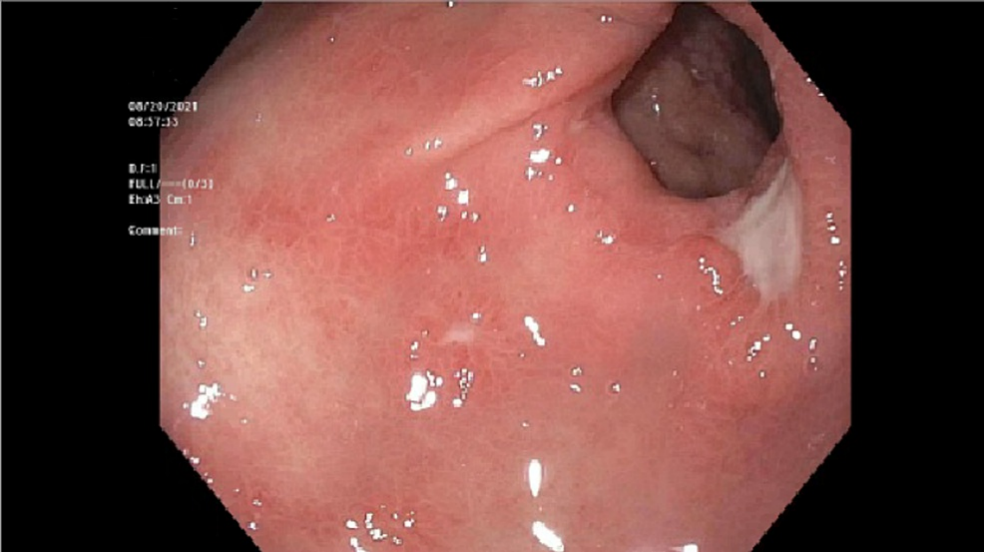
Pyloric ulceration on repeat Esophagogastroduodenoscopy (EGD).

A 72-year-old man with a past medical history of Hodgkin’s lymphoma, rectal adenocarcinoma in remission, and left total knee replacement six days prior to presentation was admitted with diffuse abdominal discomfort and no bowel movement for four days. He was initially thought to have opioid-induced constipation and was given magnesium citrate. He was found to have swelling of the left lower extremity and lower extremity doppler showed left common femoral vein deep vein thrombosis, for which he was started on Enoxaparin. However, the patient continued to have diffuse abdominal discomfort and developed vomiting. Physical exam was significant for a distended abdomen with tenderness to palpation in the epigastrium. Abdominal x-ray showed prominent gaseous distension of the stomach (Figure 1). Computed tomography of the abdomen revealed duodenal wall thickening, significant gastric distention with fluid and gastric contents, and a moderate to large stool burden without dilated bowel loops. The potassium level was within normal limits at 4.5 mmol/L. A nasogastric tube was placed for stomach decompression which revealed dark, bloody output, and the patient dropped his hemoglobin by 2 grams/dl. He was started on an intravenous proton pump inhibitor (PPI). Esophagogastroduodenoscopy (EGD) was performed the following day, which revealed a large clot in the antrum and pylorus, and the scope could not be advanced to the duodenum (Figure 2). The clot was removed partially with a polypectomy snare which revealed that it was extending into the duodenum. Limited view of the duodenal bulb showed a large blood clot occupying the lumen and causing gastric outlet obstruction. There was oozing of blood seen in the duodenal bulb but no visible vessel. The scope was unable to pass beyond the duodenal sweep due to the obstructing clot. Interventional radiology was consulted and performed a gastroduodenal artery embolization as there was no active contrast extravasation seen on the arteriogram. Subsequent EGD 72-hours later after treatment with PPI and erythromycin showed a 7mm clean-based ulcer in the pylorus and multiple media to large clean-based ulcers in the duodenal bulb and second portion (Figure 3). The patient was transitioned to an oral proton pump inhibitor with a plan for repeat endoscopy in 8-weeks for reassessment. Helicobacter pylori antibody and immunostain of the gastric biopsy were negative. 

**Figure 4:**
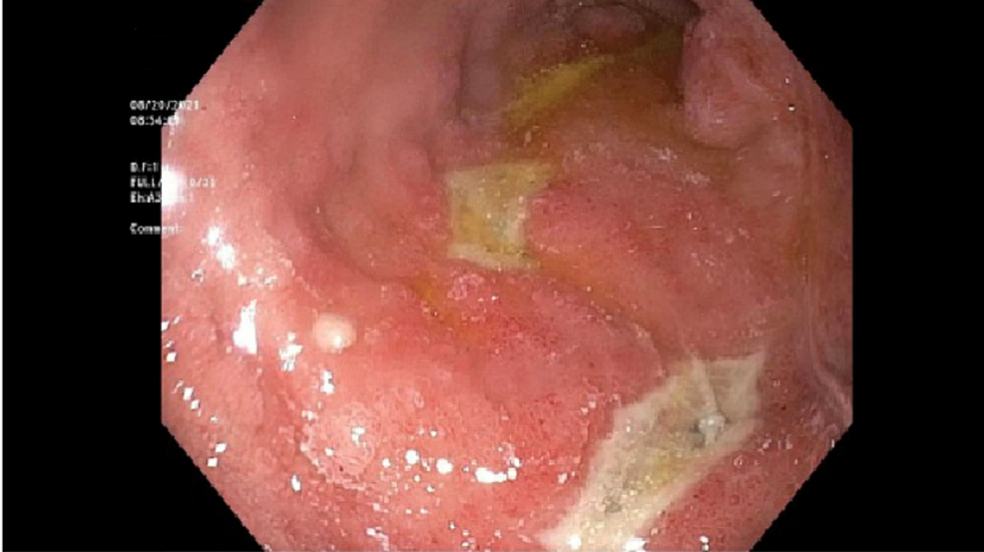
Duodenal ulcerations on repeat Esophagogastroduodenoscopy (EGD).

## Discussion

This is a rare case of a large blood clot causing gastric outlet obstruction. On initial endoscopy, the scope was unable to traverse the duodenal sweep due to the obstructing clot. It is unlikely his symptoms were due to postoperative ileus as his symptoms were delayed in onset after his knee replacement and did not improve after constipation had resolved. The patient’s pyloric and duodenal ulcers were presumably caused by nonsteroidal anti-inflammatory drug use, which he was taking for knee pain, and significant bleeding was likely due to enoxaparin. Peptic ulcer disease typically affects the pyloric channel and duodenal bulb, as in the case we describe. Gastric outlet obstruction is an uncommon complication of peptic ulcer disease and may require surgical intervention [6,7]. Diagnosis of GOO is typically made with abdominal computed tomography and confirmed with endoscopy [8]. In cases where removal of the clot is not possible endoscopically or determined to be too high risk, repeat EGD 48-72 hours later can establish the diagnosis and allow for therapeutic intervention if gastrointestinal bleeding persists. Alternatively, embolization of the gastroduodenal artery can successfully address cases of refractory or recurrent upper gastrointestinal bleeding.

## Conclusions

Gastric outlet obstruction is an uncommon complication of peptic ulcer disease. We present a case of GOO caused by a large clot attributed to peptic ulcer disease. The patient presented with abdominal pain and vomiting, and gastric distention was confirmed with imaging. A large clot was visualized in the pylorus, but the scope could not be advanced past the duodenal sweep. As the removal of the obstructing clot was deemed too high risk, the patient was treated with promotility agents that relieved the obstruction and allowed for the identification of the etiology of his upper gastrointestinal bleeding.
